# PD-L1 upregulation is associated with activation of the DNA double-strand break repair pathway in patients with colitic cancer

**DOI:** 10.1038/s41598-021-92530-3

**Published:** 2021-06-22

**Authors:** Naoya Ozawa, Takehiko Yokobori, Katsuya Osone, Chika Katayama, Kunihiko Suga, Chika Komine, Yuta Shibasaki, Takuya Shiraishi, Takuhisa Okada, Ryuji Kato, Hiroomi Ogawa, Akihiko Sano, Makoto Sakai, Makoto Sohda, Hitoshi Ojima, Tatsuya Miyazaki, Yoko Motegi, Munenori Ide, Takashi Yao, Hiroyuki Kuwano, Ken Shirabe, Hiroshi Saeki

**Affiliations:** 1 Department of General Surgical Science, Graduate School of Medicine, Gunma University, Maebashi, Gunma Japan; 2 Division of Integrated Oncology Research, Gunma University Initiative for Advanced Research (GIAR), 3-39-22 Showa-machi, Maebashi, Gunma 371-8511 Japan; 3 Department of Gastroenterological Surgery, Gunma Prefectural Cancer Center, Ohta, Gunma Japan; 4 Department of Gastroenterological Surgery, Maebashi Red Cross Hospital, Maebashi, Gunma Japan; 5 Department of Pathology Diagnosis, Maebashi Red Cross Hospital, Maebashi, Gunma Japan; 6 Department of Human Pathology, Graduate School of Medicine, Juntendo University, Bunkyo City, Tokyo, Japan

**Keywords:** Surgical oncology, Cancer microenvironment, Tumour immunology, Ulcerative colitis, Colorectal cancer

## Abstract

Ulcerative colitis (UC) is a DNA damage-associated chronic inflammatory disease; the DNA double-strand break (DSB) repair pathway participates in UC-associated dysplasia/colitic cancer carcinogenesis. The DSB/interferon regulatory factor-1 (IRF-1) pathway can induce PD-L1 expression transcriptionally. However, the association of PD-L1/DSB/IRF-1 with sporadic colorectal cancer (SCRC), and UC-associated dysplasia/colitic cancer, remains elusive. Therefore, we investigated the significance of the PD-L1/DSB repair pathway using samples from 17 SCRC and 12 UC patients with rare UC-associated dysplasia/colitic cancer cases by immunohistochemical analysis. We compared PD-L1 expression between patients with SCRC and UC-associated dysplasia/colitic cancer and determined the association between PD-L1 and the CD8+ T-cell/DSB/IRF-1 axis in UC-associated dysplasia/colitic cancer. PD-L1 expression in UC and UC-associated dysplasia/colitic cancer was higher than in normal mucosa or SCRC, and in CD8-positive T lymphocytes in UC-associated dysplasia/colitic cancer than in SCRC. Moreover, PD-L1 upregulation was associated with γH2AX (DSB marker) and IRF-1 upregulation in UC-associated dysplasia/colitic cancer. IRF-1 upregulation was associated with γH2AX upregulation in UC-associated dysplasia/colitic cancer but not in SCRC. Multicolour immunofluorescence staining validated γH2AX/IRF-1/PD-L1 co-expression in colitic cancer tissue sections. Thus, immune cell-induced inflammation might activate the DSB/IRF-1 axis, potentially serving as the primary regulatory mechanism of PD-L1 expression in UC-associated carcinogenesis.

## Introduction

Ulcerative colitis (UC) is a chronic autoinflammatory disease characterised by persistent inflammation in the colorectal mucosa, described as UC-associated dysplasia that can ultimately progress to UC-associated colorectal cancers (colitic cancer). This carcinogenetic sequence is designated as the “inflammation-dysplasia-carcinoma-sequence”^1^. Treatment of patients with UC-associated dysplasia/colitic cancer generally includes proctocolectomy with ileoanal anastomosis as metastatic or synchronic lesions are frequent^2^. In contrast, the pathogenesis of sporadic colon cancer (SCRC), which is sporadic or spontaneous colorectal cancer, is not associated with genetic factors or family history, and the associated carcinogenesis sequence is termed the “adenoma-carcinoma-sequence”^3^. Moreover, the carcinogenic sequences of colitic cancer and SCRC differ. Inflammatory responses initiated by the infiltrating immune cells and their secreted cytokines can lead to accumulation of DNA damage during UC^1^. The DNA double-strand break (DSB) repair pathway is, therefore, considered to play an important role in the carcinogenesis of both SCRC patients and rare UC patients with UC-associated dysplasia and colorectal cancers, the condition being referred to as colitic cancer^4–6^. Although DSBs are induced in the UC mucosa, rather than the normal colon mucosa^7^, the significance of DSBs in SCRC and UC-associated dysplasia/colitic cancer remains controversial^6,8,9^.

Recent advancements in immune checkpoint inhibitors (ICIs) targeting programmed cell death-1 (PD-1) and its ligand (PD-L1) have improved survival in several cancers, including SCRC, with mismatch repair deficiency (MMRD)/high microsatellite instability (MSI)^10^. The interaction between PD-1 on cytotoxic T lymphocytes (CTLs) and PD-L1 on tumour cells inhibits proliferation, survival, and effector functions in immune cells, including the secretion of inflammatory cytokines^11^. Hence, PD-L1 levels are considered a marker of sensitivity towards ICIs in several cancers^12^. Furthermore, PD-L1 expression is upregulated in UC compared with that in the uninflamed colon mucosa^13^, while PD-L1 upregulation in SCRC is associated with cancer progression and a poor prognosis^14^. However, differences in PD-L1 expression profiles between SCRC and rare UC patients with UC-associated dysplasia/colitic cancer have not yet been investigated.

Several mechanisms underlying PD-L1 expression have been reported, including release of inflammatory cytokines from several immune cells such as CTLs, DNA damage responses, Janus kinase (JAK)/signal transducer and activator of transcription (STAT) signalling, oncogenic pathways, hypoxic conditions, and microRNAs^15^. Among these, we focused on the DNA DSB repair pathway, which is important for colitis-associated carcinogenesis^16,17^. Moreover, Sato et al^18^ reported that DSBs can upregulate PD-L1, and the mechanism underlying PD-L1 expression is mediated via the activation of the DSB/interferon regulatory factor 1 (IRF-1) pathway. Indeed PD-L1 induction and overexpression by IRF-1 activation have been reported in several cancers^19,20^, with IRF-1 reportedly upregulated in UC compared with the normal colon mucosa in healthy volunteers^21^. However, limited information is available as to whether differences in PD-L1 expression profiles in SCRC and UC-associated dysplasia/colitic cancer are associated with the tumour-infiltrating immune cells/DSB/IRF-1 signalling axis in clinical samples from rare UC patients with UC-associated dysplasia/colitic cancer.

This study, therefore, aimed to investigate the associations among PD-L1 expression, immune cells, and the DSB repair pathway, and elucidate the potential mechanism underlying UC carcinogenesis. To this end, we performed immunohistochemistry for surgically resected samples from 17 SCRC patients, 12 UC patients with UC-associated dysplasia/colitic cancer, and 10 UC patients without UC-associated dysplasia/colitic cancer.

## Results

### Clinical significance of PD-L1 expression in tissues from patients with SCRC and UC-associated dysplasia/colitic cancer

**Figure 1:**
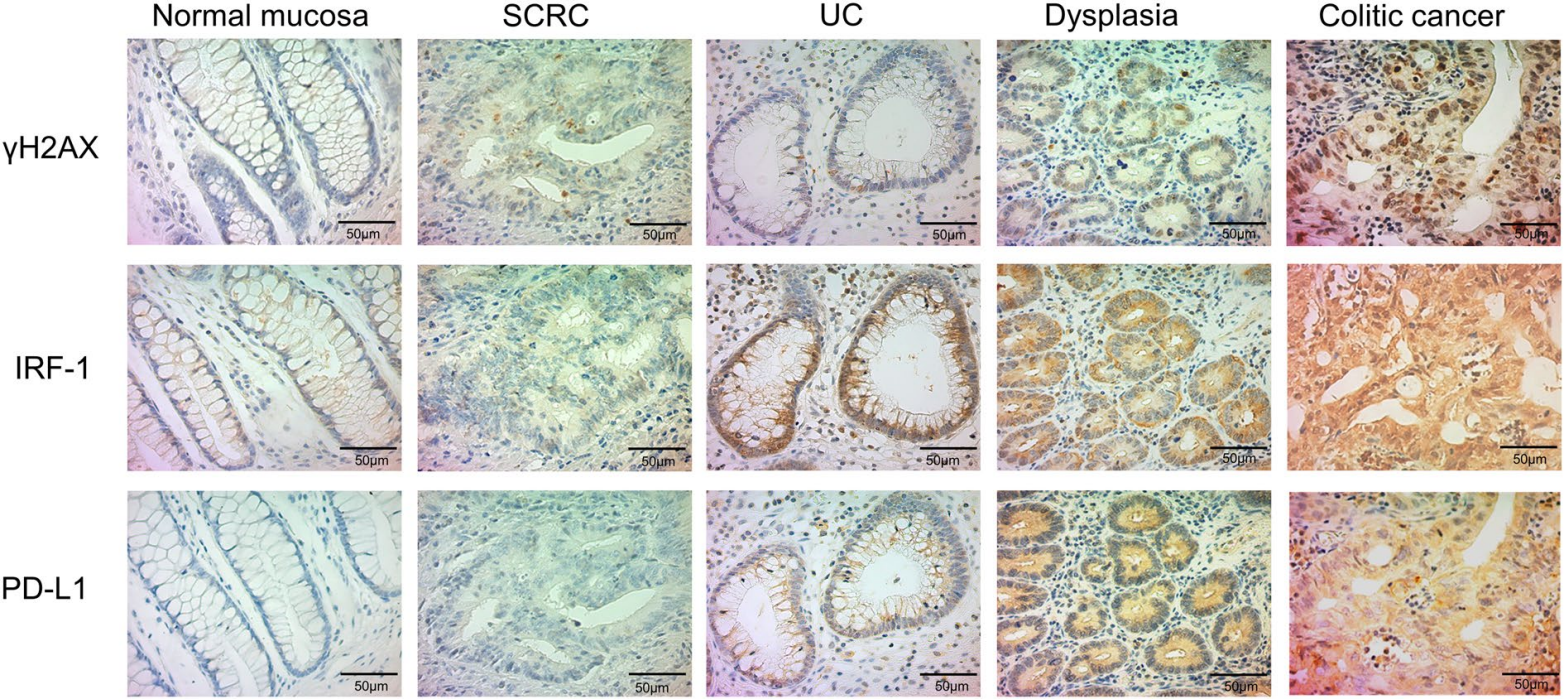
Immunohistochemistry for γH2AX, IRF-1, and PD-L1 expression in the respective colon tissues. The upper panel displays γH2AX expression in the normal mucosa, SCRC, UC, UC-associated dysplasia and colitic cancer. The middle panel displays IRF-1 expression in the normal mucosa, SCRC, UC, UC-associated dysplasia and colitic cancer. The lower panel shows the expression of PD-L1 in the normal mucosa, SCRC, UC, UC-associated dysplasia and colitic cancer. Scale bar, 50 μm (original magnification, × 400). SCRC, sporadic colorectal cancer; UC, ulcerative colitis; γH2AX, H2A.X variant histone; IRF-1, interferon regulatory factor 1; PD-L1, programmed cell death ligand 1.

Membrane and cytoplasmic PD-L1 expression was detected in colorectal tissue samples harvested from patients with SCRC and those with UC (Fig. 1). Membrane PD-L1 expression levels in tissues of UC and UC-associated dysplasia/colitic cancer were significantly higher than that in the tissues of SCRC and the corresponding normal mucosa (*P* < 0.001; Table 1). The association between PD-L1 expression and clinical and pathological factors of patients with SCRC and UC-associated dysplasia/colitic cancer is summarised in Table 2. Membrane PD-L1 expression levels differed significantly between male and female SCRC patients (*P* = 0.028; Table 2). However, the pattern of PD-L1 expression did not differ between UC mucosa with or without dysplasia/colitic cancer. The clinicopathological characteristics of UC patients with and without UC-associated dysplasia/colitic cancer are summarised in Supplementary Table 1. There was no significant difference between the expression of PD-L1 and p53/Ki-67, which are useful for the diagnosis of UC-associated dysplasia/colitic cancer, in UC patients with UC-associated dysplasia/colitic cancer (Supplementary Table 2, Supplementary Fig. 2).

**Table 1:** Expression of PD-L1 in the respective colon tissues.

Factors	PD-L1		*P* value
	Low (%)	High (%)	
^a^Normal mucosa	14 (100)	0 (0)	*P* < 0.001
^b^UC	2 (17)	10 (83)	
SCRC	11 (65)	6 (35)	
Dysplasia/colitic cancer	4 (17)	19 (83)	

*UC* ulcerative colitis, *SCRC* sporadic colorectal cancer.

^a^Normal mucosa indicates the corresponding non-cancerous tissue in sections with SCRC.

^b^UC indicates the corresponding non-cancerous tissues in sections with dysplasia/colitic cancer.

**Table 2:** Association between the expression of PD-L1 and clinicopathological factors in sporadic colorectal cancer (SCRC) and dysplasia/colitic cancer tissues.

Factors	SCRC (n = 17)		*P* value	Dysplasia/colitic cancer (n = 23)		*P* value
	PD-L1			PD-L1		
	Low	High		Low	High	
	11	6		4	19	
**Sex**						
Female	10	2	0.028	0	3	1.000
Male	1	4		2	7	
**Age**						
< 65	6	2	0.619	1	8	0.455
> 65	5	4		1	2	
**Location**						
Right	6	4	1.000	3	4	0.067
Left	5	2		1	15	
**Differentiation**						
Dysplasia/well/moderate	11	5	0.353	3	18	0.324
Poor	0	1		1	1	
**T factor**						
m, sm, mp	5	1	0.333	2	14	0.557
ss, se, si	6	5		2	5	
**N factor**						
Absent	8	4	1.000	2	17	0.125
Present	3	2		2	2	
**M factor**						
Absent	11	5	0.353	2	9	1.000
Present	0	1		0	1	
**TNM stage**						
0, I, II	8	3	0.600	0	8	0.091
III, IV	3	3		2	2	

Age, sex, M factor, and TNM stage data for dysplasia/colitic cancer are shown for a total of 12 patients, and not for the total 23 lesions analyzed in the study.

### Correlation between PD-L1 and CD8+ CTLs, DSB marker γH2AX, and IRF-1 in SCRC and UC-associated dysplasia/colitic cancer

**Figure 2:**
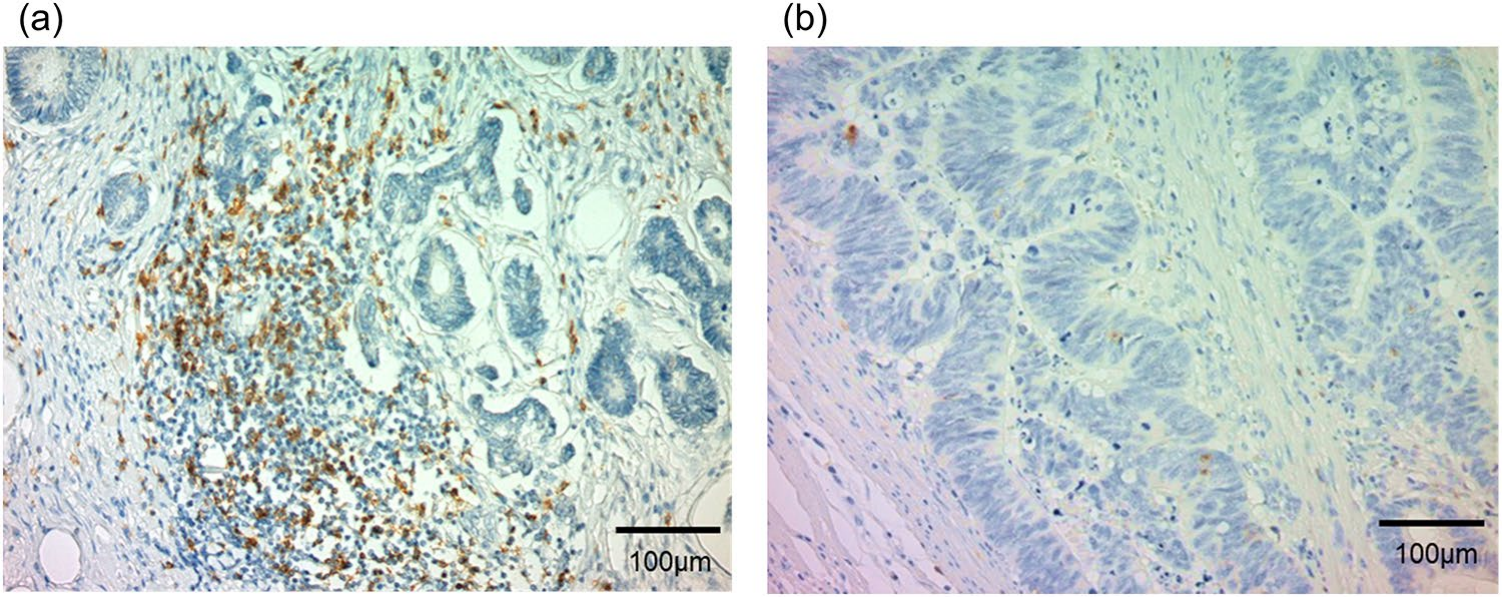
Immunohistochemistry for CD8 expression in colitic cancer and sporadic colorectal cancer. (**a**) High levels of tumoural CD8 cytotoxic T lymphocytes (CTLs) in a representative colitic cancer tissue. (**b**) Low levels of tumoural CD8 CTLs in a representative sporadic colorectal cancer (SCRC) tissue.

Figure 1 shows representative sections immunostained for γ-H2AX, IRF-1, and PD-L1 in the respective tissues. Figure 2 shows tumoural CD8 + CTLs in representative sections of SCRC and colitic cancer. PD-L1 expression in SCRC was significantly associated with IRF-1 expression (*P* = 0.035); however, no association was observed between PD-L1 expression in SCRC tissues and levels of CD8 or γH2AX (Table 3). Moreover, PD-L1 upregulation in UC-associated dysplasia/colitic cancer was significantly associated with γH2AX (*P* = 0.024) and IRF-1 (*P* = 0.002) upregulation but not with CD8 expression (*P* = 0.103; Table 3). IRF-1 upregulation in UC-associated dysplasia/colitic cancer was significantly associated with CD8 (*P* = 0.032) and γH2AX (*P* = 0.001) upregulation (Table 3). Furthermore, the expressions of CD8 as a CTL marker (*P* = 0.001), γH2AX as a DSB marker (*P* = 0.031), IRF-1 as a transcriptional factor for PD-L1 (*P* = 0.005), and PD-L1 (*P* = 0.003) in UC-associated dysplasia/colitic cancer were significantly higher than that noted for SCRC (Table 4). Meanwhile, the expression pattern of CD8, IRF-1, γH2AX, and PD-L1 between UC mucosa with and without dysplasia/colitic cancer was comparable (Supplementary Table 1). The expression patterns of target proteins in each UC patient with UC-associated dysplasia/colitic cancer are summarised in Table 5.

**Table 3:** Association between the expression of PD-L1 and IRF-1, as well as other proteins, in sporadic colorectal cancer (SCRC) and dysplasia/colitic cancer tissues.

Factors	SCRC		*P* value	Dysplasia/colitic cancer		*P* value
	PD-L1			PD-L1		
	Low	High		Low	High	
	11	6		4	19	
**CD8**						
Low	10	5	0.596	3	5	0.103
High	1	1		1	14	
**γH2AX**						
Low	4	4	0.247	2	0	0.024
High	7	2		2	19	
**IRF-1**						
Low	9	1	0.035	3	0	0.002
High	2	5		1	19	

Factors	SCRC		*P* value	Dysplasia/colitic cancer		*P* value
	IRF-1			IRF-1		
	Low	High		Low	High	
	10	7		3	20	
**CD8**						
Low	9	6	0.669	3	5	0.032
High	1	1		0	15	
**γH2AX**						
Low	4	4	0.419	3	0	0.001
High	6	3		0	20

**Table 4:** Differences in the expression of immune-related factors between sporadic colorectal cancer (SCRC) and dysplasia/colitic cancer.

Factor	SCRC	Dysplasia/colitic cancer	*P* value
**CD8**			
Low	15	8	0.001
High	2	15	
**γH2AX**			
Low	8	3	0.031
High	9	20	
**IRF-1**			
Low	10	3	0.005
High	7	20	
**PD-L1**			
Low	11	4	0.003
High	6	19

**Table 5:** Clinicopathological characteristics of patients with ulcerative colitis (UC) with UC-associated dysplasia/colitic cancer.

	Location	CD8	γH2AX	IRF-1	PD-L1	p53	Ki67	MMRD	Differentiation	T factor	N factor	M factor	Stage
**Case 1**													
UC	S	+	−	+	+	−	5.6	−	−	−	−	−	
Cancer	S	−	+	+	+	−	26.6	−	Poor	4b	0	0	IIC
**Case 2**													
UC	D	+	−	+	+	−	1.4	−	−	−	−	−	
Cancer	D	+	+	+	+	−	49.8	−	Well	1	0	0	I
Cancer	Rb	+	+	+	+	−	12.2	−	Well	2	0	0	
**Case 3**													
UC	S	−	−	+	+	+	3.0	−	−	−	−	−	
Dysplasia	Ce	−	+	+	+	+	10.2	−	−	−	−	−	
Dysplasia	A	−	+	+	+	+	3.8	−	−	−	−	−	
Dysplasia	S	−	+	+	+	+	3.5	−	−	−	−	−	
Cancer	Ce	+	+	+	+	+	28	−	Moderate	3	0	0	IIIB
Cancer	A	+	+	+	+	+	10	−	Moderate	4a	1a	0	
Cancer	S	−	+	+	+	+	44.6	−	Moderate	2	0	0	
**Case 4**													
UC	S	−	−	+	+	−	16.6	−	−	−	−	−	
Dysplasia	S	+	+	+	+	−	73.4	−	−	−	−	−	
Cancer	S	+	+	+	+	+	39.8	−	Well	2	0	0	I
**Case 5**													
UC	S	+	−	+	+	−	1.8	−	−	−	−	−	
Dysplasia	S	+	+	+	+	−	51	−	−	−	−	−	
Cancer	S	+	+	+	+	−	63.2	−	Well	4a	2a	M1b	IVB
**Case 6**													
UC	T	+	−	+	−	−	46.6	−	−	−	−	−	
Dysplasia	T	+	+	+	−	+	39	−	−	−	−	−	
Cancer	T	−	−	−	−	+	68.8	−	Moderate	3	1a	0	IIIB
**Case 7**													
UC	D	−	−	+	−	−	24	−	−	−	−	−	
Cancer	A	−	−	−	−	+	24.6	+	Poor	4a	2a	0	IIIC
Cancer	D	−	−	−	−	+	63	−	Moderate	1b	0	0	
**Case 8**													
UC	S	+	−	+	+	−	11.5	−	−	−	−	−	
Cancer	S	+	+	+	+	−	31	−	Moderate	4a	0	0	IIB
**Case 9**													
UC	Ra	−	−	−	+	−	4	−	−	−	−	−	
Cancer	Ra	+	+	+	+	−	27.3	+	Well	Tis	0	0	0
**Case 10**													
UC	Rb	+	−	+	+	−	14.1	−	−	−	−	−	
Cancer	Rb	+	+	+	+	+	95.2	+	Well	T1b	0	0	I
**Case 11**													
UC	D	+	−	−	+	−	14.1	−	−	−	−	−	
Dysplasia	D	+	+	+	+	−	48	−	−	−	−	−	
Cancer	D	+	+	+	+	−	43.3	−	Well	Tis	0	0	0
**Case 12**													
UC	RS	+	−	−	+	−	40	−	−	−	−	−	
Dysplasia	RS	+	+	+	+	+	86.2	−	−	−	−	−	0^a^

*Ce* cecum, *A* ascending colon, *T* transverse colon, *D* descending colon, *S* sigmoid colon, *RS* rectosigmoid, *Ra* upper rectum, *Rb* lower rectum.

+ , Positive or high expression of target proteins; − , Negative or low expression of target proteins.

UC indicates the corresponding noncancerous tissues in sections with dysplasia/colitic cancer.

^a^One case with no colitic cancer and only dysplasia was included in Stage 0.

### Associations among γH2AX, IRF-1, and PD-L1 expression in colitic cancer

**Figure 3:**
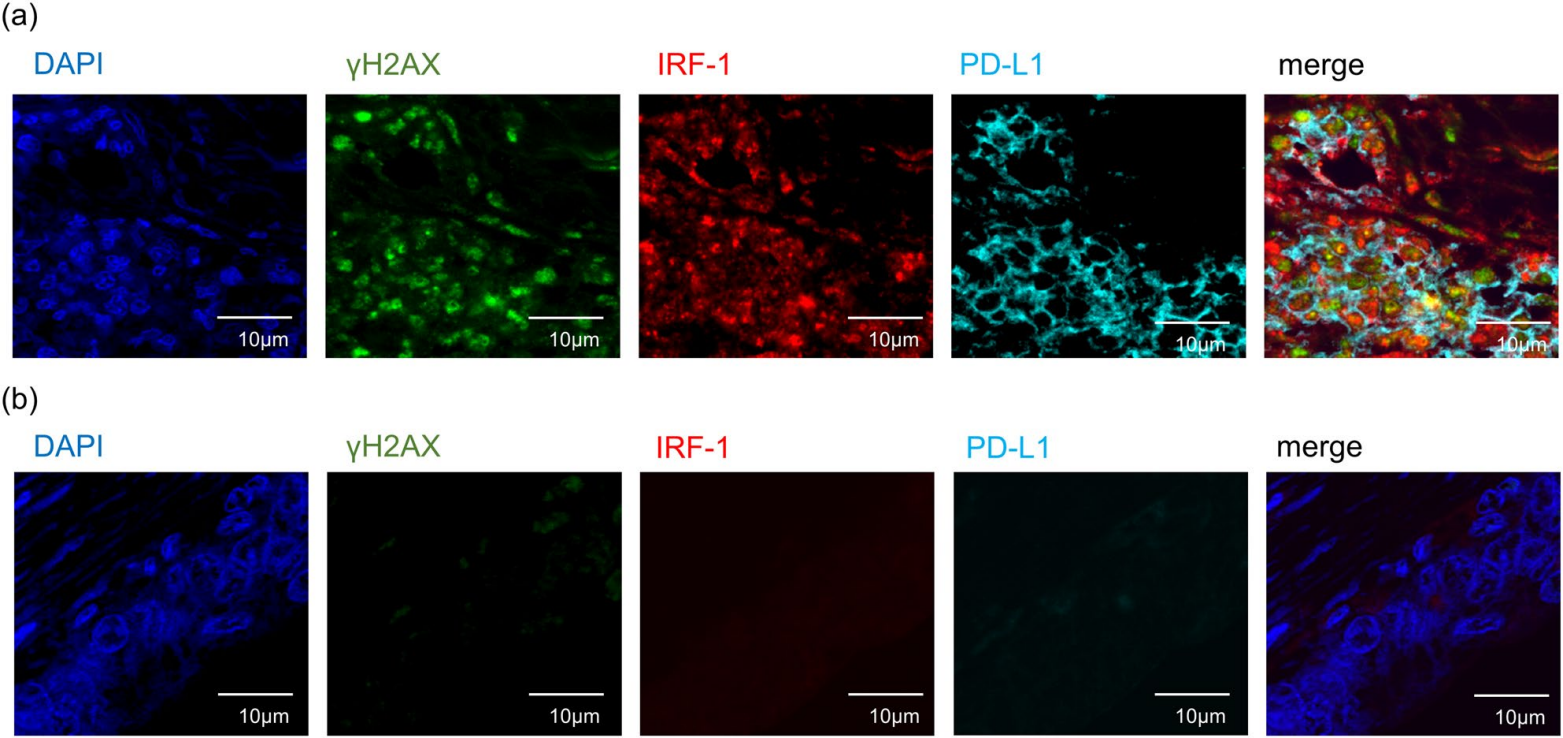
Immunofluorescence analysis for γH2AX, IRF-1, and PD-L1 expression in colitic cancer tissues. Colitic cancer tissues with high (**a**) or no (**b**) PD-L1 expression were immunostained with anti-γH2AX (green), anti-IRF-1 (red), and anti-PD-L1 (cyan) antibodies. All sections were counterstained with 4′,6-diamidino-2-phenylindole (DAPI) (blue). Scale bar, 10 μm (original magnification, ×60). γH2AX, H2A.X variant histone; IRF-1, interferon regulatory factor 1; PD-L1, programmed cell death ligand 1.

Using multicolour immunofluorescence analysis, we were able to validate the co-expression of γH2AX, IRF-1, and membrane PD-L1 in seven UC patients with colitic cancer and dysplasia. Consequently, cancer cells expressing membrane PD-L1 showed nuclear γH2AX and IRF-1 expression, in contrast to those with low PD-L1 expression, which did not show co-expression of γH2AX, IRF-1, and PD-L1 (Fig. 3). Furthermore, in SCRC tissue sections, γH2AX and IRF-1 were observed to be co-expressed in cells expressing PD-L1 as well as those that did not, suggesting that PD-L1 regulation in SCRC may not be dependent on the DNA damage response/PD-L1 signalling axis (Supplementary Fig. 3). These findings are consistent with the data presented in Table 3.

### The association of MMRD with SCRC and UC-associated dysplasia/colitic cancer

The MMRD status was not significantly different between SCRC and UC-associated dysplasia/colitic cancer (Table 5, Supplementary Table 4).

## Discussion

This study showed that PD-L1 expression levels were significantly upregulated in UC and UC-associated dysplasia/colitic cancer tissues compared with that in SCRC tissues and the corresponding non-cancerous mucosa. Moreover, PD-L1 expression was higher in tumoural CD8-positive T lymphocytes in UC-associated dysplasia/colitic cancer tissues than that in SCRC tissues. PD-L1 upregulation was found to be associated with increased expression of γH2AX (a DSB marker) and IRF-1 (a PD-L1 inducer) in clinical UC-associated dysplasia and colitic cancer tissues but not in SCRC tissues or the corresponding non-cancerous UC mucosa.

Colitic cancer is caused by UC-induced persistent chronic inflammation, and the carcinogenic sequence of colitic cancer is suggested to differ from that of SCRC^1^. Chronic inflammation in UC frequently results in DSB through the generation of reactive oxygen species, with a dysfunctional DNA damage repair system suggested to lead to carcinogenesis during UC^6–8^. However, PD-L1 is regulated by various inflammation-associated transcription factors, including STAT1/3, IRF-1, and NF-κB^19,22^. Among these, DSB-induced-IRF-1 activates PD-L1^18^. The present findings showed that PD-L1 expression in UC-associated dysplasia/colitic cancer was positively correlated with γH2AX and IRF-1 expression. These results indicated that PD-L1 expression in UC-associated dysplasia and colitic cancer was potentially associated with the activation of the DSB/IRF-1 signalling axis. Moreover, in cancer cells, PD-L1 is associated with the activation of DNA damage repair kinases such as ATR and ATM^23^. Therefore, DNA damage-induced-PD-L1 in dysplasia/colitic cancer may work not only as a mere biomarker for inflammatory DNA damage but also as a regulator of DNA repairment.

ICIs have recently attracted increasing attention as an innovative cancer treatment strategy^24^. However, the beneficial effects of ICI treatment in SCRC patients are often limited to MMRD/MSI-high cancers^25,26^, and it remains unknown whether ICIs are effective in rare cases of UC with UC-associated dysplasia/colitic cancer. Studies have reported that MMRD/MSI-high status is a promising biomarker for predicting the sensitivity to ICIs^27^, with tumours exhibiting high PD-L1/high CD8+ CTLs designated as “immune-inflamed tumours” or “hot tumours” displaying positive responses compared with ICI-resistant uninflamed tumours or ‘cold tumours’ without PD-L1 expression/CD8+ CTLs^28^. In contrast, enterocolitis is known as one of the most common adverse events associated with ICIs, and it is distinct with clinical and pathological characteristics similar to that of inflammatory bowel disease (IBD)^29,30^. A recent study showed patients with pre-existing IBD to be at an increased risk for several gastrointestinal adverse events associated with ICIs, and the safety of ICI treatment for patients with pre-existing IBD is undetermined^31^. However, TNF-α blockade treatment in patients with IBD strongly inhibits inflammation in the colorectal mucosa by suppressing several pro-inflammatory pathways, including the TNF-α pathway^32^, and immunosuppressive therapy, including TNF-α blockade and vedolizumab, which is an α4β7 integrin inhibitor blocking the migration of gut-specific lymphocytes into the gut, is administered for treating not only IBD, but also ICI-induced colitis^33,34^. Particularly, vedolizumab has been administered concurrently with ICIs in patients with ICI-induced colitis^35^. Furthermore, TNF-α blockade does not inhibit the antitumour effect of ICIs in experimentally induced melanoma^36^. Perez-Ruiz et al^37^ reported the therapeutic effect of TNF-α blockade against ICI induced-colitis in tumour-bearing mouse models. Several studies have demonstrated that ICIs can be administered without occurrence of serious adverse events in a small number of patients with IBD^38–40^, and Frohne et al^41^ reported that the combination of vedolizumab and ICI could be successfully used in treating a patient having metastatic melanoma with active Crohn's disease and suppressed Crohn’s disease flares and ICI-induced colitis. This study showed that PD-L1 and IRF-1 expression levels, as well as CD8+ CTLs in UC-associated dysplasia/colitic cancer increased significantly compared with that in the SCRC samples, suggesting that UC-associated dysplasia/colitic cancer, rather than SCRC, was potentially associated with the “hot tumour” phenotype. Furthermore, patients with melanoma having high IRF-1 expression levels exhibit a significantly higher sensitivity towards ICI treatment than those with low IRF-1 expression^42^. These results suggested that ICI treatment may be more effective in patients with UC-associated dysplasia/colitic cancer with distant metastases after total colectomy who are not at a risk for ICI-induced colitis than in patients with SCRC, because patients with UC-associated dysplasia/colitic cancer exhibit clinical characteristics including favourable ICI responses, “hot tumour” immune phenotypes, including high PD-L1/high CD8+ CTLs, and high IRF-1 expression. Moreover, the combinatorial treatment with ICIs and immunosuppressive therapy such as TNF-α blockade-vedolizumab may suppress ICI-induced colitis in patients with UC-related dysplasia/colitic cancer and exert antitumour effects.

Nonetheless, our study has several limitations. First, this was a retrospective study with a small patient cohort because UC-associated dysplasia and colitic cancer are rare types of cancer; this may have introduced a bias in our results. Hence, prospective studies with a larger patient cohort may be challenging for clearly elucidating the UC carcinogenesis mechanisms, considering the rarity of the disease. Currently, biological data on UC with UC-associate dysplasia/colitic cancer have been obtained from small-scale clinical cohort studies. This study provides further valuable insights into the importance of PD-L1 expression in patients with UC with UC-associated dysplasia/colitic cancer. Second, this study discussed the potential use of ICIs for colitic cancer treatment; however, we only enrolled patients that did not receive ICI treatment targeting the PD-1/PD-L1 signalling axis. Therefore, future studies are required to evaluate the potential of DSB/IRF-1/PD-L1 as a biomarker for rare cases of UC-associated dysplasia/colitic cancer to be treated with ICIs. Lastly, only three target markers were assessed, which may not be sufficient to clarify the complexity of the interactions between PD-L1 and the DSB repair response. Therefore, in the future we plan to examine the importance of and relationship among these three markers in an experimental animal model^43^ of inflammatory colorectal carcinogenesis.

In conclusion, this study showed that PD-L1 expression was significantly higher in UC and UC-associated dysplasia/colitic cancer than in the normal mucosa and SCRC. Furthermore, PD-L1 expression was significantly associated with high levels of tumoural CD8+ CTLs, γH2AX as a DSB marker, and IRF-1 as a transcriptional factor for PD-L1 in UC-associated dysplasia/colitic cancer compared with SCRC. Therefore, our findings suggested that immune cell induced-chronic inflammation might activate the DSB/IRF-1 signalling axis, which might serve as the primary regulatory mechanism for regulating PD-L1 expression in UC carcinogenesis.

## Methods

### Patients and samples

Twelve patients (nine male and three female) with UC, who underwent surgical resection for UC-associated dysplasia/colitic cancer at Gunma University Hospital (Maebashi, Gunma, Japan), Maebashi Red Cross Hospital (Maebashi, Gunma, Japan), and Gunma Prefectural Cancer Center (Ohta, Gunma, Japan) between 1999 and 2014, were included in this retrospective study. The median age of the patients was 54 years (range, 37–76 years). One patient only had UC-associated dysplasia. Three patients harboured two or more tumours, and all dysplastic and cancerous lesions were evaluated. All dysplastic lesion samples were obtained from patients with high-grade dysplasia, while patients with low-grade dysplasia were not included in the study. Additionally, 17 SCRC patients (12 male and five female) who underwent partial colectomy at the Gunma University Hospital between 1999 and 2014 were randomly selected and included in the study. Ten control UC patients (seven males and three females) who underwent surgical resection for reasons other than UC-associated dysplasia/colitic cancer at Gunma University Hospital were also included in the analysis. Supplementary Tables 1 and 2 summarise the clinical characteristics of the patients. Supplementary Table 3 shows the characteristics of the clinical course in UC patients with UC-associated dysplasia/colitic cancer. We evaluated the severity of UC as previously reported^44^. For an accurate pathological diagnosis of dysplastic and cancerous lesions among patients with UC, all histological cancer tissue sections were evaluated by a specialised pathologist, Dr. Yao T (Department of Human Pathology, Juntendo University Graduate School of Medicine). This study conformed to the tenets of the Declaration of Helsinki and was approved by the Institutional Review Board for Clinical Research at the Gunma University Hospital, Maebashi, Gunma, Japan (approved number: HS2018-092). Owing to the retrospective nature of this study, need for informed consent was waived, and participants were given the opportunity to decline participate in this study via opt-out method. All methods were performed in accordance with relevant guidelines and regulations.

### Immunohistochemistry

Paraffin-embedded blocks of all surgically resected specimens obtained from the patients were cut into 4 µm thick sections and mounted on glass slides. Sections were deparaffinised with xylene and dehydrated in alcohol. Endogenous peroxidase was inhibited using 0.3% H_2_O_2_/methanol for 30 min at approximately 26 °C. After rehydration through a graded ethanol series, antigen retrieval was performed using Immunosaver (Nisshin EM, Tokyo, Japan) at 98–100 °C for 45 min, and PD-L1 was retrieved using Universal HIER antigen retrieval reagent (Abcam, ab208572) at 120 °C for 20 min in an autoclave. Nonspecific binding sites were blocked through incubation with Protein Block Serum-Free (Dako, Carpinteria, CA, USA) for 30 min. Thereafter, sections were probed with primary anti-PD-L1 (Abcam, 28-8 Rabbit mAb, 1:200), anti-γH2AX (Abcam, 9F3, Mouse mAb, 1:200; DSB marker)^45^, anti-IRF-1 (Abcam, EPR 18301, Rabbit mAb, 1:300), anti-CD8 (Dako C8/144B, Mouse mAb, 1:100), anti-p53 (DAKO, DO-7, Mouse mAb, 1:100), anti-Ki67 (DAKO, MIB-1, Mouse mAb, 1:300), anti-MLH1 (DAKO, ES05, Mouse mAb), anti-MSH2 (DAKO, FE11, Mouse mAb), anti-MSH6 (DAKO, EP49, Rabbit mAb), and anti-PMS2 (DAKO, EP51, Rabbit mAb) antibodies at 4 °C for 24 h. The slides were then stained using rabbit-specific IHC polymer detection kit HRP/DAB (Abcam, ab209101) containing the secondary antibody for PD-L1-stained sections, and with the Histofine Simple Stain MAX-PO (Multi) Kit (Nichirei, Tokyo, Japan) for other markers, in accordance with the manufacturer’s instructions. The chromogen 3,3′-diaminobenzidine tetrahydrochloride was applied as a 0.02% solution, containing 0.005% H_2_O_2_ in ammonium acetate-citrate acid buffer (50 mM, pH 6.0). Finally, nuclear counterstaining was performed using Mayer’s haematoxylin solution. In the negative control sections, primary antibodies were replaced with phosphate-buffered saline in 0.1% bovine serum albumin, thus confirming a lack of staining.

### Assessment of immunohistochemistry staining

Immunostained sections were evaluated by two experienced researchers blinded to the clinical data. We evaluated tumour cells and non-cancerous cells displaying membrane PD-L1 staining as positive when at least 1% of the cells were stained^46^. The staining intensity for γH2AX was scored as follows: 0, no staining; 1+, weak staining; 2+, moderate staining; and 3+, strong staining. The percentage of nuclear-stained cells was determined by examining three sections with the highest staining intensity. The percentage of nuclear γH2AX staining was scored as follows: 0, no staining; 1+, 1–25%; 2+, 26–50%; and 3+, 51–100%. The final score was defined as the percentage score multiplied by the intensity score (0, 1+, 2+, 3+, 4+, 6+, and 9+). Nuclear immunoreactivity of γH2AX was scored as 0–4+ and 6–9+, which were defined as low and high nuclear expression, respectively^47^. We evaluated nuclear and cytoplasmic staining for IRF-1 using almost the same method as that for γH2AX described above. The staining percentage for IRF-1 was scored as follows: 0, no staining; 1+, 1–25%; 2+, 26–75%; and 3+, 76–100%^48^. The final score was defined as the percentage score multiplied by the intensity score (0, 1+, 2+, 3+, 4+, 6+, and 9+). IRF-1 immunoreactivity was scored as 0–4+ and 6–9+ , which were defined as low and high expression, respectively. Supplementary Fig. 2 shows the representative low or high expression of γH2AX, IRF-1, and PD-L1 in SCRC and UC-associated dysplasia/colitic cancer tissues. Tumoural CD8-positive CTLs, defined as CD8 cells infiltrating the cancer stroma, were scored as low or high when the mean number of cells in a microscopic field at ×200 magnification was < 200 or ≥ 200, respectively^49^.

p53‑positive cells were defined as previously reported^50^. The Ki-67 labelling index was scored as a percentage of positively stained cells. MMRD was defined as the complete absence of the expression of at least one mismatch repair protein (MLH1, MSH2, MSH6, or PMS2).

### Multicolour immunofluorescence staining for PD-L1, γH2AX, and IRF-1

Multicolour immunofluorescence staining was performed for seven patients with UC on a total of seven colitic cancer and two dysplasia tissue sections, and three SCRC tissue sections using a PerkinElmer Opal kit (Catalogue# NEL810001KT, PerkinElmer, Hopkinton, MA, USA) in accordance with the manufacturer’s instructions. γH2AX staining was visualised using the Opal 520 Fluorophore, IRF-1 staining with the Opal 690 Fluorophore, and PD-L1 staining with the Opal 570 Fluorophore. All sections were then counterstained with 4′,6-diamidino-2-phenylindole (DAPI) and examined under an FV10i-LIV system (Olympus Life Science, Tokyo, Japan).

### Statistical analysis

The JMP Pro 14.0 software package (SAS Institute Inc., Cary, NC, USA) was used for statistical analysis. Mann‒Whitney U test and Fisher's exact test were performed to analyse the associations among PD-L1 expression, immune cells, and the DSB repair pathway. All differences were considered statistically significant at *P* < 0.05.

## Supplementary Information

Supplementary Information.

## Data Availability

The datasets generated during and/or analysed during the current study are available from the corresponding author on reasonable request.
