# Supplementary material for: PD-L1 upregulation is associated with activation of the DNA double-strand break repair pathway in patients with colitic cancer
Source: Sci Rep. 2021 Jun 22;11:13077. doi: 10.1038/s41598-021-92530-3 (PMC8219733; doi:10.1038/s41598-021-92530-3)
Supplement: Supplementary file 1 — Supplementary Information. [file 41598_2021_92530_MOESM1_ESM.docx]

**PD-L1 upregulation is associated with activation of the DNA double-strand break repair pathway in patients with colitic cancer**

Naoya Ozawa^1^, Takehiko Yokobori^2*^, Katsuya Osone^1^, Chika Katayama^1^, Kunihiko Suga^1^, Chika Komine^1^, Yuta Shibasaki^1^, Takuya Shiraishi^1^, Takuhisa Okada^1^, Ryuji Kato^1^, Hiroomi Ogawa^1^, Akihiko Sano^1^, Makoto Sakai^1^, Makoto Sohda^1^, Hitoshi Ojima^3^, Tatsuya Miyazaki^4^, Yoko Motegi^4^, Munenori Ide^5^, Takashi Yao^6^, Hiroyuki Kuwano^1^, Ken Shirabe^1^, and Hiroshi Saeki^1^

^1^ Department of General Surgical Science, Graduate School of Medicine, Gunma University, Maebashi, Gunma, Japan

^2^ Division of Integrated Oncology Research, Gunma University Initiative for Advanced Research (GIAR), Maebashi, Gunma, Japan

^3^ Department of Gastroenterological Surgery, Gunma Prefectural Cancer Center, Ohta, Gunma, Japan

^4^ Department of Gastroenterological Surgery, Maebashi Red Cross Hospital, Maebashi, Gunma, Japan

^5^ Department of Pathology Diagnosis, Maebashi Red Cross Hospital, Maebashi, Gunma, Japan

^6^ Department of Human Pathology, Graduate School of Medicine, Juntendo University, Bunkyouku, Tokyo

* Corresponding author: Takehiko Yokobori

Division of Integrated Oncology Research, Gunma University Initiative for Advanced Research (GIAR), 3-39-22 Showa-machi, Maebashi 371-8511, Gunma, Japan; Tel: +81-027-220-8224; Fax: +81-027-220-8230; Email: [bori45@gunma-u.ac.jp](mailto:bori45@gunma-u.ac.jp)

**Supplementary Tables**

**Supplementary Figures**

**
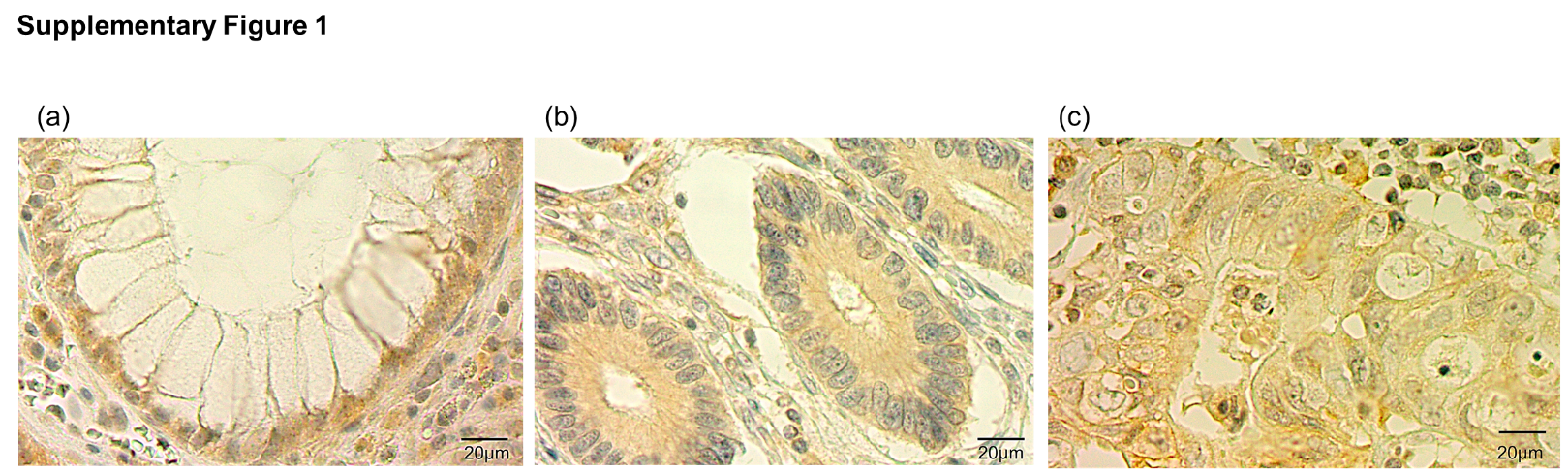
**

**Supplementary Figure 1. High-magnification images showing immunohistochemical staining of PD-L1 in representative UC and UC-associated dysplasia/colitic cancer tissues.**

(a) High-magnification image showing PD-L1 immunohistochemical staining in UC tissue. (b) High-magnification image showing PD-L1 immunohistochemical staining in UC-associated dysplasia tissue. (c) High-magnification image showing PD-L1 immunohistochemical staining in colitic cancer tissue. Scale bar, 20 μm. PD-L1, programmed cell death ligand 1; UC, ulcerative colitis.

**
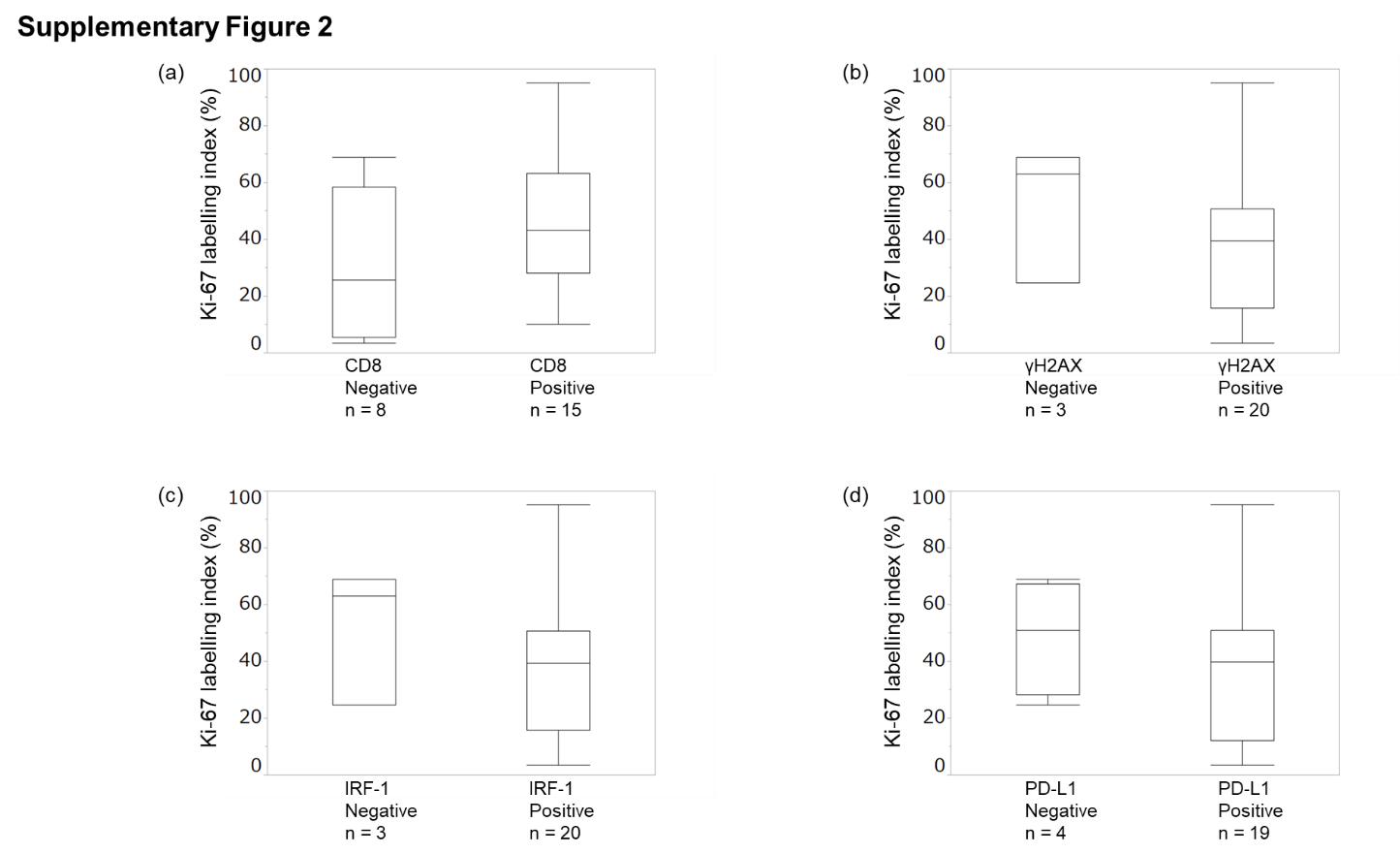
**

**Supplementary Figure 2.** **Box and whisker plots showed a relationship between the Ki-67 labelling index and the expression of CD8, γH2AX, IRF-1, and PD-L1 in dysplasia/colitic cancer tissues.**

Box and whisker plots showed a relationship between the Ki-67 labelling index and the expression of (a) CD8, (b) γH2AX, (c) IRF-1, and (d) PD-L1 in dysplasia/colitic cancer tissues. Outliers and the distribution interval were illustrated. There was no significant difference in the relationship between the Ki-67 labelling index and the expression of these proteins in dysplasia/colitic cancer tissues. γH2AX, H2A.X variant histone; IRF-1, interferon regulatory factor 1; PD-L1, programmed cell death ligand 1.

**
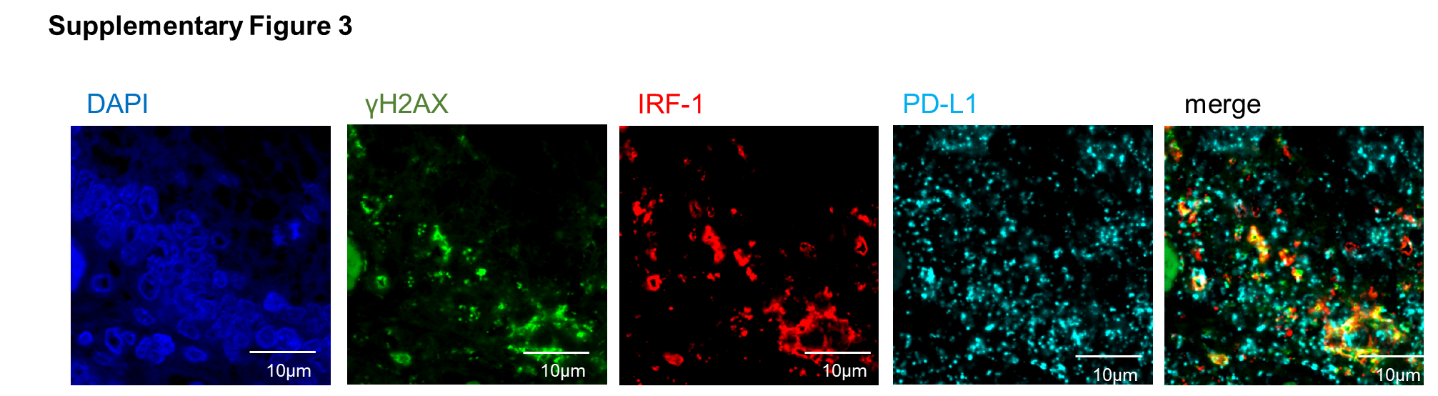
**

**Supplementary Figure 3. Immunofluorescence analysis for γH2AX, IRF-1 and PD-L1 expression in SCRC tissues.**

Sporadic colorectal cancer (SCRC) tissues with high PD-L1 expression levels were immunostained with anti-γH2AX (green), anti-IRF-1 (red), and anti-PD-L1 (cyan) antibodies. All sections were counterstained with 4',6-diamidino-2-phenylindole (DAPI) (blue). Scale bar, 10 μm; original magnification, ×60. γH2AX, H2A.X variant histone; IRF-1, interferon regulatory factor 1; PD-L1, programmed cell death ligand 1.

**
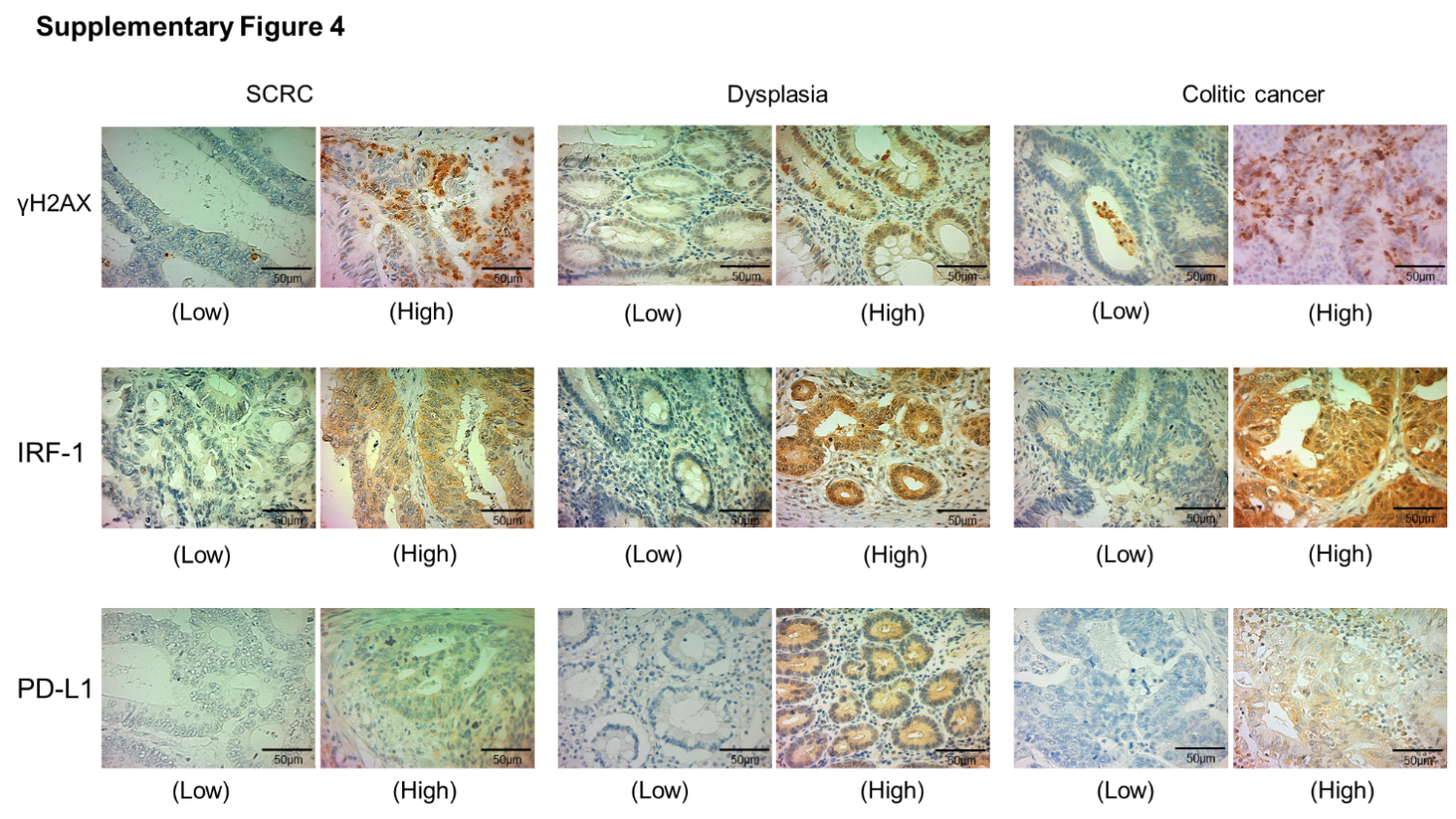
**

**Supplementary Figure 4. Low or high expression of γH2AX, IRF-1, and PD-L1 in the representative tissues of SCRC, UC-associated dysplasia, and colitic cancer.**

The upper panel displays the low or high γH2AX expression in SCRC, UC-associated dysplasia, and colitic cancer. The middle panel displays the low or high IRF-1 expression in SCRC, UC-associated dysplasia, and colitic cancer. The lower panel displays the low or high expression of PD-L1 in SCRC, UC-associated dysplasia, and colitic cancer. Scale bar, 50 μm; original magnification, ×400. SCRC, sporadic colorectal cancer; UC, ulcerative colitis; γH2AX, H2A.X variant histone; IRF-1, interferon regulatory factor 1; PD-L1, programmed cell death ligand 1.
